# Clinical application of live biotherapeutic products in infectious diseases

**DOI:** 10.3389/frmbi.2024.1415083

**Published:** 2024-07-30

**Authors:** Bhagyashri D. Navalkele, Teena Chopra

**Affiliations:** ^1^ Division of Infectious Diseases, Emory University School of Medicine, Atlanta, GA, United States; ^2^ Division of Infectious Diseases, Wayne State University/Detroit Medical Center, Detroit, MI, United States

**Keywords:** live biotherapeutic product, live biotherapeutic product in infectious diseases, live biotherapeutic product for *Clostridioides difficile*, live biotherapeutic product for multidrug resistant organisms, live biotherapeutic product for viral infection, live biotherapeutic product for bacterial infection

## Abstract

Live biotherapeutics products (LBP) are a novel range of therapeutic options in medicine. In this review, authors discuss basic composition and mechanism of action of LBP, provide a comprehensive focused overview of published *in vitro* and *in vivo* studies on efficacy of LBP for prevention and treatment of infectious diseases such as viral (HIV, COVID-19), bacterial (*C.difficile* infection, bacterial vaginosis, multi-drug resistant organisms) and fungal (Candida) organisms. This review should be of interest to clinicians to understand the broad application of LBP in infectious diseases world beyond recurrent *C.difficile* infection and to researchers on unexplored prospects of LBP and the need for further investigation in this emerging field to improve its clinical application.

## Introduction

The human microbiome refers to a variety of microorganisms ranging from bacteria, yeasts, viruses, protozoa, archaea, and eukaryotic microbes colonizing the body (Aroniadis and Grinspan, 2024). These microbial communities inhabit the oral, nasopharyngeal, gut, genitourinary and skin lining. The interaction between the host and microbial communities is known to play an important role in human health and diseases. Gut microbiota, the most abundant of all microbiomes, has been associated with a wide variety of metabolic and immune mediated diseases like inflammatory bowel diseases, obesity, cancer, neurological disorders. Since birth, multiple factors such as diet, environment, genetics, antibiotics influence the composition of human gut microbiome (Aroniadis and Grinspan, 2024). Understanding this microbial community and complex host-microbiome interaction has been challenging. Recently, metagenomic studies have led to better understanding of composition of the gut microbiome and impact of alteration in gut microbiome aka dysbiosis on subsequent development of diseases and disorders. As a result, microbiome engineering is now an emerging novel technique designed to alter microbial composition or its activity for therapeutic purposes to help restore normal health function. Live biotherapeutic products (LBPs) are such an example of genetically engineered microbes used for diagnostic and therapeutic purposes.

The US Food and Drug Administration (Food and Drug Administration, 2016) defines LBPs as “a biological product that 1) contains live organisms such as bacteria; 2) is applicable to the prevention, treatment, or cure of a disease or condition of human beings; and 3) is not a vaccine”. Recombinant LBP is defined as “a live biotherapeutic product composed of microorganisms that have been genetically modified through the purposeful addition, deletion, or modification of genetic material”. In contrast to LBPs, prebiotics, probiotics and synbiotics are commonly used as functional foods and dietary supplements, without need for regulatory approval process and lack robust clinical trials data on efficacy against any specific disease prevention and treatment (**Table 1**).

**Table 1:** Comparison between prebiotics, probiotics, synbiotics and live biotherapeutic products.

	Prebiotics (Davani-Davari et al., 2019)	Probiotics (O’Toole and Cooney, 2008)	Synbiotics (Swanson et al., 2020)	Live biotherapeutic products (Rutter et al., 2022)
Definition	“A non-digestible food ingredient that beneficially affects the host by selectively stimulating the growth and/or activity of one or a limited number of bacteria in the colon, and thus improves host health”	“Live microorganisms that, when administered in adequate amounts, confer a health benefit on the host”	“A mixture comprising live microorganisms and substrate(s) selectively utilized by host microorganisms that confers a health benefit on the host”	“Biological product that 1) contains live organisms such as bacteria; 2) is applicable to the prevention, treatment, or cure of a disease or condition of human beings; and 3) is not a vaccine”
Use	Dietary supplement	Dietary supplement	Drug, food or supplement	Medicinal use for range of diseases such as *C.difficile* infection, colitis, inflammatory bowel diseases, cancer, phenylketonuria
Examples	Fructo-oligosaccharides (FOS), Galacto-oligosaccharides (GOS), Lactose, Inulin-enriched FOS	*Lactobacillus spp, Bifidobacterium* spp.*, Saccharomyces, Bacillus* spp.*, Escherichia coli, Enterococci, Weissella* spp.	*Streptococcus thermophilus* in combination with FOS/GOS/Inulin-enriched FOS	*E.coli* Nissle 1917, *Lactococcus lactis*, *Salmonella typhi* VXM01
Regulatory approval requirement	No	No	No	Yes

### Host microbiome and association with infection

The human microbiome can resist pathogens from developing infection via direct and indirect pathways. Microbiome can result in direct killing of pathogens, without host involvement, by producing metabolites, toxins and depriving pathogens of essential nutrients (Zhang et al., 2021). For example, vaginal microbiome predominantly consists of Lactobacillus species which produces lactic acid (Kwon and Lee, 2022). Acidic vaginal environment causes inhibition of growth of harmful bacteria like *E.coli*, *N.gonorrhea* and *C.trachomatis*. Lactic acid can also prevent viral infection such as inactivation of HIV surface charge preventing its attachment to host cell. Indirectly, host microbiome can activate innate and adaptive immune responses and stimulate anti-pathogen metabolite production to resist pathogenic infection. Common skin colonizer like *S.epidermidis* can induce CD8 T cells providing antimicrobial protection and helping with tissue repair (Zheng et al., 2020). In a similar fashion to host microbiome, LBPs can be a single microbial strain, or a community of microbial strains designed to interact with host ecosystem using innate microbial properties or through genetic modification (Heavey et al., 2022). These engineered LBPs can function by inhibiting growth of pathogenic organisms or toxins, stimulating production of beneficial molecules to prevent cancer, to modifying metabolism and mucosal immune system to decrease inflammation (Tan et al., 2020). A study performed on human colonic explants pretreated with a single-strain live biotherapeutic product (SS-LBP) of *Bacillus velezensis* strain ADS024 followed by *C.difficile* toxin exposure demonstrated protease secretion by ADS024 resulting in lower toxin B levels and toxin-mediated cell apoptosis (O’Donnell et al., 2022). Thus, SS-LBP has potential for protecting against colonic mucosal damage and preventing recurrent CDI post standard antimicrobial treatment.

## Use of live biotherapeutic products in infectious diseases

### *Clostridioides difficile* infection

*Clostridioides difficile* is one of the most common healthcare-associated infections with high recurrence rates and accounting for 15%-20% all-cause mortality. Exposure to antimicrobial therapy results in gut microbiota dysbiosis and higher primary bile acid concentration increasing the risk for *Clostridioides difficile* infection (CDI) and its recurrence. Treatment guidelines for CDI recommend oral antimicrobial therapy options such vancomycin, fidaxomicin and fecal microbiota transplant (FMT) for recurrent CDI (rCDI). However, antimicrobial CDI treatment has failed to show sustained response in preventing rCDI and the potential spread of pathogenic organisms to recipients via FMT remains a concern.

Live biotherapeutic products have been extensively studied in treatment and prevention of recurrent CDI (Pettit et al., 2024). Microbiota based suspension has been demonstrated to enhance normal gut microbiota (commensal *Bacterioidia* and *Clostridia*) and reduce harmful microbes (*Gammaproteobacteria*, *Bacilli* and *Erysipelotrichia*), thus restoring normal gut flora as well as beneficial secondary bile acid concentrations (Orenstein et al., 2023).

RBX2660 containing a consortium of spore and non-spore forming fecal microbiota, live-jslm (Rebyota), has been studied in a phase 3 clinical trial (PUNCH CD3) administered as a single-dose rectal administration to adults 24-72hrs after completion of standard antibiotic therapy for rCDI (Khanna et al., 2022). Compared to placebo, RBX2660 demonstrated safety and 70.6% efficacy in preventing rCDI at week 8 with sustained effects for up to 6 months post treatment (Lee et al., 2023). Similar findings were observed in prevention of rCDIs in high-risk elderly patients treated with RBX2660 (Tillotson et al., 2022). Fecal microbiota spores, live-brpk or VOS (Vowst Oral Spores) containing suspension of Firmicutes spore forming colonies has been studied in phase 3 ECOSPOR III clinical trial to assess CDI recurrence after completion of anti-CDI therapy (Sims et al., 2023). Treatment of adult patients with oral dose of four VOS capsules for 3 days showed lower rCDI rates compared to placebo and sustained response for up to 24 weeks post treatment. Similar efficacy was seen in older adults through week 8.

A non-frozen oral encapsulated LBP (RBX7455) containing processed fecal microbiota-based suspension of RBX2660 from a single donor successfully completed phase 1 clinical trial demonstrating its safety and efficacy in preventing recurrent CDI (rCDI) in adults for up to 6 months post treatment (Fischer and Ray, 2024).

The phase 2 clinical trial of encapsulated high-dose oral formulation (10 capsules daily for 14 days) containing 8 well-characterized nontoxigenic nonpathogenic commensal *Clostridia* strains (VE303) has demonstrated efficacy in preventing rCDI in adults and primary CDI in high-risk elderly patients (Louie et al., 2023).

Treatment with nontoxigenic strain *C. difficile*-M3 (NTCD-M3) spores possess the ability to colonize gastrointestinal tract inhibiting proliferation of pathogenic *C.difficile*. A phase 2b clinical trial performed with daily oral formulation of NTCD-M3 spores for 7 days showed 95% efficacy in preventing rCDI for 6-weeks post treatment (Gerding et al., 2015).

## Gastrointestinal infections other than *C. difficile*

Studies performed in *in vitro* and *in vivo* models have demonstrated antibacterial activity of recombinant human lactoferrin produced by *Lactobacillus casei* (Tan et al., 2020). Similarly, *Lactococcus lactis* is known to secrete anti-enterococcal peptide capable of inhibiting enterococcal growth exerting antimicrobial activity against *E.faecalis* and multidrug resistant *E.faecium* strains.

Traveler’s diarrhea is caused by enterotoxin produced by Enterotoxigenic *E.coli* strain. Paton et al. constructed nonpathogenic *E.coli* CWG308 strain expressing glycosyltransferase genes from *Neisseria meningitidis* or *Campylobacter jejuni* resulting in production of modified lipopolysaccharide (mLPS) lacto-N-neotetraose (Paton et al., 2005). Structural similarity of mLPS to Shiga toxin’s natural receptor resulted in binding and neutralization of enterotoxin in both human and porcine *in vitro* models as well as *in vivo* rabbit ligated ileal loops.

To combat cholera disease, *E.coli* Nissle 1917 constructed to express cholera autoinducer 1 resulted in reducing cholera toxin binding and intestinal colonization of infant mouse model along with significant growth inhibition of *V.cholerae* (Kelly et al., 2009; Duan and March, 2010). Similarly, *E.coli* Nissle 1917 strain engineered to produce antimicrobial peptides Microcin H47 in presence of tetrathionate, an indicator of intestinal inflammation, has demonstrated ability to inhibit growth of Salmonella species.

### Multidrug-resistant organisms

Antibiotic exposure, infections with multidrug-resistant organisms (MDROs) can result in alteration of gut microbiota (Panzer et al., 2024). Patients harboring MDROs are known to develop long-term gut colonization with these organisms increasing risk for reinfection and transmission to others. In view of lack of robust data on decolonization strategies for MDROs, use of LPB to decolonize gut without risk for dysbiosis is a promising alternative (Ducarmon et al., 2021).

Engineered *E.coli* Nissle 1917 modified to overproduce microcin I47 has demonstrated antimicrobial activity against multiple MDRO, with highest efficacy against Enterobacteriaceae (Sonnenborn, 2016; Charbonneau et al., 2020). Preclinical animal model study demonstrated significant decline in intestinal concentration of carbapenem-resistant *K.pneumonaie* without change in native microbiome after seven days of daily oral LBP treatment (Mortzfeld et al., 2022). Genetically engineered *E.coli* Nissle 1917 strains with ability to produce dispersin B, an antibiofilm enzyme, has demonstrated decline in *Pseudomonas aeruginosa* concentrations in animal models and reduction in Enterococcal and Salmonella species in murine models through secretion of antimicrobial peptides (Hwang et al., 2017).

### Coronavirus disease 2019 infection

Coronavirus disease 2019 (COVID-19) caused by the virus SARS-CoV-2 has accounted for high morbidity and mortality. Currently, omicron variants continue to circulate in communities worldwide causing mild to severe life-threatening infections including breakthrough infections. Outside of antivirals, LBP has been studied as potential treatment options for COVID-19.

Oral LL-37 is an LBP generated by combination of *Lactococcus lactis* (drug delivery agent) and Human antimicrobial peptide LL-37 (Zhao et al., 2023). In general, Human LL-37 is known to exert antiviral activity by disrupting lipid envelope, causing cell death. Studies have demonstrated LL-37 prevents binding of SARS-CoV-2 spike protein to host cell receptor ACE2 reducing risk for COVID-19 infection. In an investigator-initiated prospective open-labeled randomized case-control single-center clinical trial, authors compared the effect of oral LL-37 vs placebo (*L.lactis*) on negative conversion time (NCT) of SARS CoV-2 nucleic acid test. Adult patients with mild Omicron BA.5 infection received oral LL-37 (n=129) or placebo (n=109) for seven days. Treatment with oral LL-37 resulted in significantly reduced NCT when initiated within 6 days of infection without any safety concerns. Additional studies are needed to demonstrate if early initiation of oral LL-37, reducing SARS CoV-2 viral load, could potentially have other beneficial roles such as shorter duration of clinical symptoms and lower risk for long COVID.

### Respiratory tract infections

Viral respiratory tract infections are known to cause many diseases from common cold infections to severe pneumonia. Other than influenza and COVID-19, there is lack of robust data on antiviral treatment recommendations for other viruses. Respiratory microbiome is known to resist viral infections with antipathogenic properties, immune activation and maintenance of epithelial barrier integrity. *In vitro* topical testing has demonstrated *Lactobacillaceae* strains to directly inhibit respiratory viruses and stimulate interferon regulatory pathways. Further *in vitro* analysis of three strains *Lacticaseibacillus casei* AMBR2, *Lacticaseibacillus rhamnosus* GG and *Lactiplantibacillus plantarum* WCFS1 has shown to have combined anticytopathogenic effects against respiratory syncytial virus, influenza viruses and human coronaviruses-229E. The combination of these three strains suspended in oil delivered in form of a throat spray was investigated in healthy volunteers (Spacova et al., 2023). Study confirmed microbiome throat spray resulted in immune activation and colonization of throat with all 3 *Lactobacillacea* strains for 30 minutes post application. Further clinical trials are needed to assess the safety and efficacy of these products for practical use.

### Urinary tract infections

Urinary tract infections (UTIs) can range from uncomplicated cystitis to pyelonephritis. It is the one of the most common bacterial infections in the world accounting for high-cost burden to the healthcare industry. Frequent use of antimicrobials for treatment of UTIs has resulted in a rise in antimicrobial resistance and limitation in available effective treatment options. Live biotherapeutic products have been studied as potential options for treatment and prevention of UTIs.

Normal urogenital flora is known to have protective factors which prevent ascending infections. Less UTIs were observed after vaginal application of *Lactobacillus crispatus* causing restoration of native microbial flora. Asymptomatic bacteriuria (ASB) *E.coli* strain has been shown to possess bacterial interference properties by inhibition of uropathogens via unknown mechanism to prevent UTIs (Rudick et al., 2014). Additionally, intravesicular or intravaginal ASB *E.coli* instillation has shown to possess analgesic activity similar to intravesicular lidocaine. Clinical studies performed in spinal cord injury patients, neurogenic bladder patients with chronic catheterizations, and recurrent UTIs showed significantly lower UTIs after inoculation of ASB *E.coli* strain 83972. Furthermore, ASB *E.coli* strain 83972 has shown comparable results to ciprofloxacin in reducing NU14 bacteriuria in mice models. ASB E.coli strain also demonstrated lower UTI allodynia in infections with pathogenic *E.coli* as well as other common gram negative and gram-positive uropathogens. Overall, LBPs have therapeutic benefit for UTIs as well as symptomatic relief in interstitial cystitis patients, reducing the risk for unnecessary antibiotic use in these patient population.

### Human immunodeficiency virus

Every year more than 30,000 new human immunodeficiency virus (HIV) infections are diagnosed in the United States. Currently, prevention of HIV relies mostly on safe sex and needle use practices. Pre-exposure HIV prophylaxis with oral tenofovir-emtricitabine based antiretrovirals and long-acting cabotegravir injectable agents are available. Adherence to medications, risk of viral resistance and breakthrough infections remains a concern.

Normal vaginal flora has been known to possess beneficial properties resisting urogenital infections such as sexually transmitted diseases and HIV. Lactobacilli strains, commonly found in 17%-41% of vaginal flora, produce hydrogen peroxide and lactic acid reducing the risk of acquiring sexually transmitted infections and HIV-1.

Recombinant LBP MucoCept is a genetically engineered *Lactobacillus* strain, *L. jensenii* 1153-1666 designed to prevent HIV infection in women by secreting modified HIV-1 inhibitor protein, cyanovirin-N preventing mucosal viral entry (Lagenaur et al., 2015). Study in rhesus macaque model inoculated with vaginal MucoCept showed 63% reduction in acquisition of chimeric simian-HIV and six-fold reduction in animal viral loads with breakthrough infections. Potent, stable, easy-to-use, rapidly disintegrating vaginal tablets of MucoCept successfully colonized 83% of macaques after 21 days. Clinical trials are underway to test novel once-monthly MucoCept vaginal rings as a non-antiretroviral option for prevention of HIV-1 infection in women.

### Bacterial vaginosis

Bacterial vaginosis (BV) is one of the most common vaginal infections in reproductive age women associated with high recurrence rate and increased risk for HIV infection due to genital inflammation and epithelial barrier disruption. The condition is known to occur due to imbalance in bacterial flora causing overgrowth of anaerobic bacteria and reduction in hydrogen-peroxide-producing Lactobacilli species, which normally predominate vaginal flora. Among Lactobacilli species, *L.crispatus* is known to be predominant with anti-inflammatory properties and suppression of proinflammatory bacterial species reducing the risk for HIV acquisition. Standard antibiotic treatment of BV has been shown to reduce proinflammatory cytokines, however it increases Interferon-gamma-induced protein (IP)-10 associated with HIV acquisition risk and does not restore beneficial Lactobacillus species. Studies with Lactobacillus species-based probiotics have been unsuccessful in lowering genital inflammation and BV recurrence.

Live biotherapeutic containing *L.crispatus* strain CTV-05 (LACTIN-V) has been investigated in phase 2 clinical trial post standard topical metronidazole treatment of BV (Armstrong et al., 2022). Compared to placebo, vaginal application of LACTIN-V resulted in lower rates of recurrent BV sustained up to 3-months post last treatment dose. Study findings showed LACTIN-V lowered concentrations of genital pro-inflammatory cytokines and chemokines, suppressed inflammatory bacterial vaginosis-associated bacteria and diminished epithelial barrier disruption. LACTIN-V is a promising treatment option for preventing recurrent BV and an appealing non-antiretroviral option to prevent HIV acquisition in women.

### Candidiasis

Candidiasis is a fungal infection caused by the Candida species, predominantly *C.albicans*, a common part of normal human microbial flora. Opportunistic infections can range from mild thrush to life-threatening invasive diseases. Treatment options are limited to 3 antifungal classes and emerging resistance remains a concern. *Lactobacillus casei rhamnosus* Lcr35 (Lcr35) is known to possess anti-pathogenic properties against *Candida albicans* by preventing its adhesion to epithelial cells (Poupet et al., 2019). *In vitro* testing on Caco-2 monolayer, an enterocyte-like cell line, failed to show any effect of Lcr35 on *C.albicans* growth or biofilm formation. However, *in vivo* study on *C.albicans* infected Caenorhabditis elegans worm showed protective efficacy of Lcr35. Mechanistic study demonstrated Lcr35 mediated activation of host’s immune response (DAF-16/Forkhead Box O transcription factor, upregulation of p38 MAP Kinase signaling pathway) and antifungal response as likely mechanism of action against *C.albicans*. Study findings provide insight into exploring potential therapeutic options in future for treatment of Candidiasis.

## Discussion

Infectious diseases are caused by pathogenic bacteria, fungi, viruses or parasites. Management of infectious diseases ranges from symptomatic treatment to antimicrobial therapy for days to weeks. Currently, widespread use of antimicrobial therapy has resulted in a rise in antimicrobial resistance and its spread. Moreover, challenges encountered with lack of effective treatment options have become a rising concern. Live biotherapeutic products containing modified normal microbiota demonstrating ability to restore microbial flora and kill pathogenic organisms are ideal non-antibiotic treatment strategy to resolve ongoing challenges.

Preclinical and clinical studies with LBP have been successful in prevention and treatment of many infectious diseases (**Table 2**). Most robust data is available in prevention of rCDIs. Faecal microbiota transplantation has been successful in preventing recurrent CDIs. However, reports of extended-spectrum beta-lactamase-producing *E.coli* infection and Shiga-toxin producing *E.coli* infection have been reported post FMT (Park and Seo, 2021). Incomplete screening of donor stool for pathogenic organisms has raised safety concerns related to FMT. Additionally, obesity and immune-mediated disorders have been linked with donor microbiome engraftment. In contrast, the FDA Center for Biologic Evaluation and Research (CBER) regulates the development of LBP including clinical trial studies to ensure its safety and efficacy (Cordaillat-Simmons et al., 2020). Facilities conducting manufacturing, processing and packaging of LBP operate under regulations of current good manufacturing processes to provide safe and reliable products. Recently, recombinant LBPs Rebyota and Vowst Oral Spores have been FDA approved for use in prevention of recurrent CDI.

**Table 2:** Clinical application of live biotherapeutic products in Infectious Diseases.

Indication	Live Biotherapeutic Product	Mode of delivery	Clinical evidence
Prevention of recurrent *C.difficile* infection	Fecal microbiota spores, live-brpk or VOS (Vowst Oral Spores)	Oral capsules	Phase 3 ECOSPOR III clinical trial, FDA approved
	RBX2660, consortium of spore and non-spore forming fecal microbiota, live-jslm (Rebyota)	Rectal administration	Phase 3 clinical trial (PUNCH CD3), FDA approved
	RBX7455, fecal microbiota-based suspension from single donor	Oral capsules	Phase 1 clinical trial
	VE303, nontoxigenic nonpathogenic commensal Clostridia strains	Oral capsules	Phase 2 clinical trial
	Nontoxigenic *C.difficile*-M3 spores (NTCD-M3)	Oral capsules	Phase 2b clinical trial
Prevention and treatment of bacterial gastrointestinal infections (other than *C.difficile*)	Lactobacillus casei, Lactobacillus lactis, nonpathogenic *E.coli* CWG308, *E.coli* Nissle 1917	Unknown	Preclinical models
Gastrointestinal decolonization of Enterobacteriaceae including multidrug resistant organisms	*E.coli* Nissle 1917	Oral capsules	Preclinical models
Treatment of COVID-19 infection	Human LL-37 (*Lactococcus lactis* and human antimicrobial peptide LL-37)	Oral capsules	Prospective open-labeled randomized case-control single center clinical trial
Prevention and treatment of respiratory tract viral infections	*Lacticaseibacillus casei* AMBR2, *Lacticaseibacillus rhamnosus* GG and *Lactiplantibacillus plantarum* WCFS1	Throat spray	Phase 1 clinical trial
Prevention and treatment of urinary tract infections	*Lactobacillus crispatus*, Asymptomatic bacteruria *E.coli* strain 83972	Intravaginal or intravesicular application	Preclinical models
Prevention of HIV	*L. jensenii* 1153-1666 (MucoCept)	Vaginal tablets	Preclinical models
Prevention and treatment of bacterial vaginosis	*Lactobacillus crispatus* strain CTV-05 (LACTIN-V)	Vaginal application	Phase 2 clinical trial
Treatment of Candidiasis	*Lactobacillus casei rhamnosus* Lcr35 (Lcr35)	Unknown	Preclinical models

In general, clinical development of LBP is a rigorous process of conducting genome sequencing of preferred clinical bacterial strain, detecting for any transferrable resistant genes, restricting replication of the microorganism in the human body and determining its biodistribution outside the site of action (Charbonneau et al., 2020). The major challenge in LBP development remains the lack of full understanding on interaction between host, microbiota, and environmental factors (Rouanet et al., 2020) Oral administration of LBP has been the most common mode of delivery thus far and considered safe and convenient for patient use. However, numerous physiological challenges encountered in human gastrointestinal tract including but not limited to stomach acids, bile acids, various enzymes, immune cells, native colonic flora, chemical environment, peristalsis, mucosal and epithelial regeneration can alter LBP functions (Heavey et al., 2022). Thus, determining appropriate delivery methods and dosage forms of LBP for treatment and prevention of diseases remains a mystery (Pot and Vandenplas, 2021).

Regardless, LBPs have tremendous potential for clinical application in both infectious and non-infectious diseases fields. The availability of safe and effective alternative non-antibiotic treatment options for management of infectious diseases will be a game changer. Use of LBPs for treatment and prevention of commonly encountered infections such as UTIs, respiratory viral infections, rCDI, and other GI tract infections can have significant healthcare and economic impact. With the current challenges of antimicrobial resistance, LBP are essentially the holy grail for management of infectious diseases.

Further studies are necessary to overcome ongoing challenges. To improve the development of LBP, application of multi-omics (e.g. genomics, transcriptomics, proteomics, metabolomics) studies are essential for better understanding of host-LBP-microbiome interactions, to identify effective delivery methods and dosage forms of LBP for preventative and therapeutic purposes (Heavey et al., 2022). Many of the current studies have focused on the use of recombinant *E.coli* and *Lactobacillus* strains. Additional exploratory work will be crucial to discover other beneficial microbes in the vast human microbiota and their potential clinical applications.

In conclusion, with rising antimicrobial resistance and limited antimicrobial treatment armamentarium, LBPs are essential and promising non-antimicrobial treatment options for management of infectious diseases. Advances in omics strategies and genetic engineering technology are necessary to perfect these products and use them in the real-world setting.

## Author contributions

BN: Writing – original draft, Writing – review & editing, Investigation, Resources. TC: Writing – review & editing, Supervision.
